# Involvement of *let-7* microRNA for the therapeutic effects of Rhenium-188-embedded liposomal nanoparticles on orthotopic human head and neck cancer model

**DOI:** 10.18632/oncotarget.11666

**Published:** 2016-08-29

**Authors:** Liang-Ting Lin, Chun-Yuan Chang, Chih-Hsien Chang, Hsin-Ell Wang, Shih-Hwa Chiou, Ren-Shyan Liu, Te-Wei Lee, Yi-Jang Lee

**Affiliations:** ^1^ Department of Biomedical Imaging and Radiological Sciences, National Yang-Ming University, Taipei, Taiwan; ^2^ Department of Medical Research and Education, Taipei Veterans General Hospital, Taipei, Taiwan; ^3^ Isotope Application Division, Institute of Nuclear Energy Research, Taoyuan, Taiwan; ^4^ Institute of Clinical Medicine, School of Medicine, National Yang-Ming University, Taipei, Taiwan; ^5^ Institute of Pharmacology, National Yang-Ming University, Taipei, Taiwan; ^6^ Biophotonics and Molecular Imaging Research Center (BMIRC), National Yang-Ming University, Taipei, Taiwan

**Keywords:** ^188^Re-liposome, HNSCC, orthotopic tumor model, microarray analysis, let-7 microRNA

## Abstract

Human head and neck squamous cell carcinoma (HNSCC) is usually treated by surgical resection with adjuvant radio-chemotherapy. In this study, we examined whether the radiopharmaceutical ^188^Re-liposome could suppress the growth of HNSCC followed by an investigation of molecular mechanisms. The orthotopic HNSCC tumor model was established by human hypopharyngeal FaDu carcinoma cells harboring multiple reporter genes. The drug targeting and therapeutic efficacy of ^188^Re-liposome were examined using *in vivo* imaging, bio-distribution, pharmacokinetics, and dosimetry. The results showed that ^188^Re-liposome significantly accumulated in the tumor lesion compared to free ^188^Re. The circulation time and tumor targeting of ^188^Re-liposome were also longer than that of free ^188^Re in tumor-bearing mice. The tumor growth was suppressed by ^188^Re-liposome up to three weeks using a single dose treatment. Subsequently, microarray analysis followed by Ingenuity Pathway Analysis (IPA) showed that tumor suppressor *let-7* microRNA could be an upstream regulator induced by ^188^Re-liposome to regulate downstream genes. Additionally, inhibition of *let-7i* could reduce the effects of ^188^Re-liposome on suppression of tumor growth, suggesting that *let-7* family was involved in ^188^Re-liposome mediated suppression of tumor growth *in vivo*. Our data suggest that ^188^Re-liposome could be a novel strategy for targeting HNSCC partially via induction of *let-7* microRNA.

## INTRODUCTION

Head and neck squamous cell carcinoma (HNSCC) is the sixth most common cancer type by incidence globally. More than 600,000 new cases are diagnosed annually [1]. Importantly, the five-year survival rate of patients is less than 50%. Although combined external beam radiation therapy (EBRT) and chemotherapy effectively contribute to the outcome of HNSCC tumor killing [2], the target radionuclide therapy (TRT) has also been reported using monoclonal antibodies conjugated radionuclides [3]. However, it is prerequisite to confirm the antigens of tumors before using TRT, because surface targets would express differently in various types of HNSCC.

Liposome is a nanoscale spherical vector with a lipid bilayer membrane structure that can encapsulate either water-soluble drugs within the aqueous cavity or lipid-soluble drugs attached in the membrane [4]. Polyethylene glycols are usually used to decorate liposomes for prolonged circulation in the bloodstream [5]. The radiopharmaceuticals made by conjugating liposome and radionuclide, such as ^186^Re and ^188^Re, are considered advantageous for concomitant diagnosis and therapy of diseases in deep tissues through the enhanced permeability and retention (EPR) effect [6, 7]. The pharmaceutical efficacy of liposome conjugated ^188^Re has been examined in animal models of human colorectal cancer, glioblastomas, esophageal cancer, and lung cancer [8–11]. However, ^188^Re-liposome has not been investigated in human HNSCC, although intratumoral injection of ^186^Re-liposome was reported to suppress the growth of xenograft HNSCC tumors [12, 13].

MicroRNAs (miRNAs) are small non-coding RNAs that inhibit the translational initiation by binding to the 3′ untranslated region (UTR) of their targets. Several biologic processes such as cell cycle control, apoptosis, and stem cell differentiation have been implied to be governed by miRNAs [14]. The family of *let-7* is one of the first two discovered microRNAs in *C. elegans*. They are encoded by various gene clusters but sharing the same seeding region, and are reported highly expressed in mammals [15]. There are 13 different members in human *let-7* family [16]. *Let-7* family has been shown to participate in the control of cellular pluripotency, proliferation and differentiation and has been defined as a tumor suppressor gene [17, 18]. Currently, it is unclear whether ^188^Re-liposome could induce *let-7* or other tumor suppressive microRNA for therapeutic effects or not.

In this study, we established the orthotopic HNSCC xenograft tumor model to evaluate the biodistribution, pharmacokinetics, drug accumulation, and therapeutic efficacy of the PEGylated ^188^Re-liposomal nanoparticles. Additionally, we performed a microarray analysis and compared the global gene expressive profiles of HNSCC tumors with and without ^188^Re-liposome treatment. It was found that the tumor suppressive *let-7* miRNA could be up-regulated by ^188^Re-liposome and involved in mediating the therapeutic efficacy of this radiopharmaceutical. The association between radionuclide based therapy and change of microRNA expression was discussed.

## RESULTS

### Establishment of orthotopic human HNSCC tumor-bearing mice model

**Figure 1:**
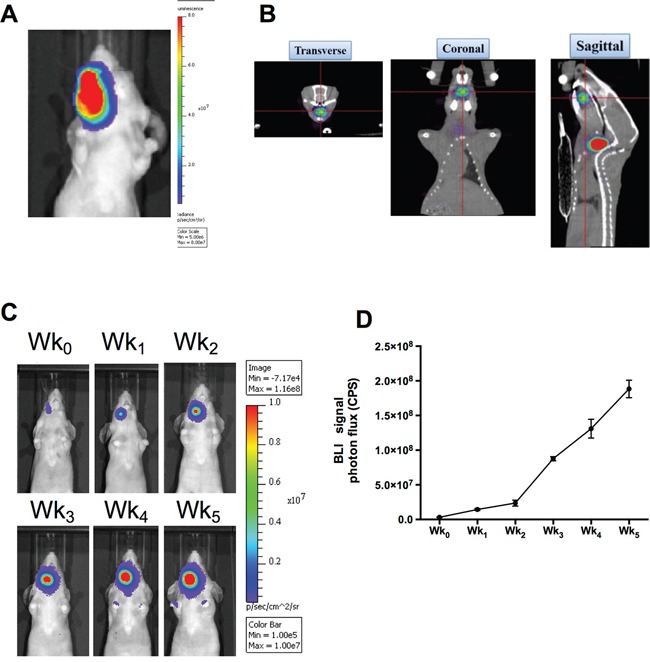
Reporter gene imaging for tracking the growth of human HNSCC in xenograft tumor model **A.** Orthotopic implantation of FaDu-3R cells at buccal cavity and detection of formed tumor using the IVIS optical imaging system; **B.** *in vivo* detection of orthotopic HNSCC xenograft tumor with HSV1-tk activity using nanoSPECT/CT and 3D reconstruction of the images. The ^123^I-FIAU was used as the radiotracer; **C.** use of IVIS optical imaging system to monitor the tumor growth weekly. Wk_0_ indicated the initial imaging acquired immediately after tumor implantation; **D.** quantification of the photon flux to plot the tumor growth curve *in vivo*.

The FaDu-3R cells were sorted and characterized for the expression of GFP, fLuc and HSV1-tk reporter genes (Supplementary Figure 1). The FaDu-3R cells were then implanted into nude mice at the positions of buccal cavity. The tumors were palpable after five weeks of implantation, and the bioluminescent imaging (BLI) indicated the tumor location in this orthotopic tumor model (Figure 1A). ^123^I-FIAU is a substrate of HSV1-tk reporter gene that can be used for nanoSPECT/CT imaging of tumor position *in vivo* (see Materials and Methods). The 3D image of orthotopic FaDu-3R tumor was reconstructed via transverse, coronal and sagittal angles to confirm the result of BLI imaging (Figure 1B). The tumor growth was monitored every week after FaDu-3R cells were implanted into the buccal cavity using BLI imaging. The BLI signals were gradually increased up to five weeks (Figure 1C). The photons influx was semi-quantified and showed that tumor growth rate was accompanied by the increment of BLI signals (Figure 1D).

### Imaging of ^188^Re-liposome accumulation in orthotopic HNSCC tumor model

**Figure 2:**
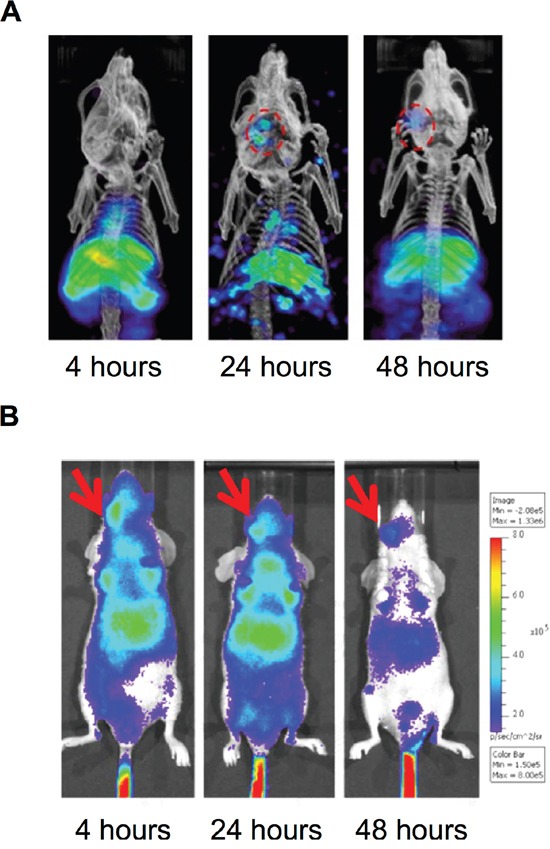
Accumulation of PEGylated ^188^Re-liposome in orthotopic HNSCC tumor lesion **A.** γ-rays emission of ^188^Re-liposome was detected in the HNSCC tumor lesion *in vivo* at different time points using nanoSPECT/CT imaging, Circles represented the region of interest (ROI); **B.** β particles emission caused Cerenokov bioluminescent imaging by ^188^Re-liposome was detected using the IVIS optical imaging system. The arrows indicated positions of implanted tumors.

We next investigated whether PEGylated ^188^Re-liposome could suppress human HNSCC in the orthotopic tumor model. The ^188^Re-liposome was intravenously injected into the tumor-bearing mice, which were imaged using nanoSPECT/CT at 4, 24, and 48 hours after administration. The reconstructed 3D nanoSPECT/CT images showed that most apparent accumulation of ^188^Re-liposome in the lower oral cavity was after 24 hours of injection, but the signal was reduced after 48 hours of injection (Figure 2A). Because ^188^Re also emits high-energy β particles, we also detected the accumulation of ^188^Re-liposome by the principle of Cerenkov luminescent imaging (CLI) that is more sensitive than radioactive signals [27]. Using the bioluminescent imaging system, the signal seemed to be detected at right neck side of tumor-bearing mouse after ^188^Re-liposome injection for 4 hours, although the background signals were also extremely strong (Figure 2B). These data demonstrated that that PEGylated ^188^Re-liposome could accumulate in tumor site after whole-body circulation.

### The biodistribution, pharmacokinetics, and dosimetric measurement of ^188^Re-liposome administrating in HNSCC xenograft tumor model

**Figure 3:**
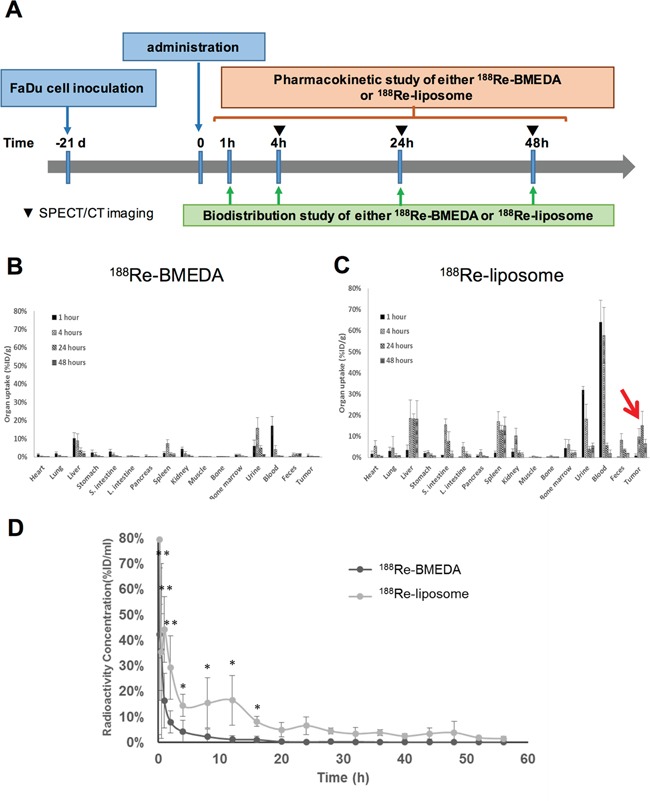
Determination of the biodistribution and pharmacokinetics of PEGylated ^188^Re-liposome used in the orthotopic HNSCC tumor model **A.** Illustration of timeline for execution of biodistribution and pharmacokinetics analysis after administration of ^188^Re-liposome or ^188^Re-BMEDA into the tumor-bearing mice; **B.** biodistribution of ^188^Re-BMEDA; **C.** biodistribution of ^188^Re-liposome. The arrow indicated the accumulation of ^188^Re-liposome in tumors. N=5 for each time point; **D.** comparison of pharmacokinetics of ^188^Re-BMEDA and ^188^Re-liposome by detecting the radioactivity in blood. N=5 for each group.

The accumulating efficiency of ^188^Re-liposome in tumors was further investigated using biodistribution analysis. The timeline of tumor inoculation and drug administration followed by biodistribution and pharmacokinetics analysis was illustrated (Figure 3A). The procedures of biodistribution analysis have been described in the Materials and Methods. The results showed that the radioactivity was rapidly washed out in the ^188^Re-BMEDA injected mice (Figure 3B). In contrast, ^188^Re-liposome could highly circulate in animal bodies up to 48 hours (Figure 3C). Notably, ^188^Re-liposome was significantly accumulated in tumor lesions, which reached the highest value (15.14 %ID/g) and marked with the highest tumor-to-muscle ratio (70.84%) after 24 hours of injection (Figure 3C). We next evaluated the pharmacokinetics of these radionuclides in the tumor-bearing mice. The ^188^Re-BMEDA and ^188^Re-liposome (100 μCi/100 μl) were separately injected into mice. The blood samples were then collected by tail vein puncture with microliter capillary tubes. The results demonstrated that the ^188^Re-liposome exhibited a slower pace of clearance and a longer retention time than those injected with ^188^Re-BMEDA (Figure 3D). The area under curves (AUC) and mean retention time (MRT) calculated from the time activity curves were also compared between ^188^Re-BMEDA and ^188^Re-liposome (Table 1). These data suggested that ^188^Re-liposome exhibited longer circulation time than controlled ^188^Re-BMEDA *in vivo*. Furthermore, we estimated the radiation dose in humans based on the results of biodistribution data in mice using the OLINDA/EXM software (Table 2). Interestingly, although the position of HNSCC was close to brain, the tumor-to-brain ratio could be up to 5.06-fold (within a 1-g sphere model). The doses of trapped ^188^Re-liposome in different size of spheroid tumor mass were also estimated (Supplementary Figure 2). Liver, spleen and heart walls were major organs with higher absorbed dose than tumor, which was in part consistent with previous studies [8].

**Table 1:** The pharmacokinetic parameters of orthotopic HNSCC animal model

Parameter	Unit	^188^Re-BMEDA	^188^Re-Liposome
***C_max_***	%ID/ml	38.77	79.31
**Cl**	ml/h	2.40±0.804	0.180±0.005
**AUC^a^_(0→∞)_**	h·%ID/ml	45.84±18.875	571.96±17.797
**MRT**^b^**_(0→∞)_**	hours	2.85±2.42	18.36±2.334

a AUC: Area Under Curve

b MRT: Mean Retention Time

**Table 2:** Dosimetric estimation of ^188^Re-liposome in human organs according to the results of biodistribution analysis in tumor-bearing mice^a^

Target Organ	Absorbed dose (mSv/MBq)
Adrenals	7.25 × 10^−2^
Brain	7.04 × 10^−2^
Breasts	7.00 × 10^−2^
Gallbladder Wall	7.43 × 10^−2^
LLI Wall^b^	1.19 × 10^−1^
Small Intestine	3.54 × 10^−1^
Stomach Wall	8.94 × 10^−2^
ULI Wall^b^	7.26 × 10^−2^
Heart Wall	5.56 × 10^−1^
Kidneys	1.54 × 10^−1^
Liver	6.74 × 10^−1^
Lungs	7.63 × 10^−2^
Muscle	3.47 × 10^−3^
Pancreas	4.42 × 10^−2^
Red Marrow	1.10 × 10^−1^
Osteogenic Cells	1.63 × 10^−1^
Skin	6.93 × 10^−2^
Spleen	4.89 × 10^−1^
Testes	6.99 × 10^−2^
Thymus	7.14 × 10^−2^
Thyroid	7.01 × 10^−2^
Urinary Bladder Wall	2.47 × 10^−1^
Tumor (1g)^c^	3.56 × 10^−1^

a The radiation dosimetry was converted from the biodistribution of ^188^Re-liposome in 0.025kg mice to 70kg male adults.

b LLI: Lower Large Intestine; ULI: Upper Large intestine.

c The tumor absorbed dose is obtained using the sphere model, and the unit is mGy/MBq because no organ weighting factor is available for tumor.

### Evaluation of therapeutic efficacy of ^188^Re-liposome on HNSCC tumor model

**Figure 4:**
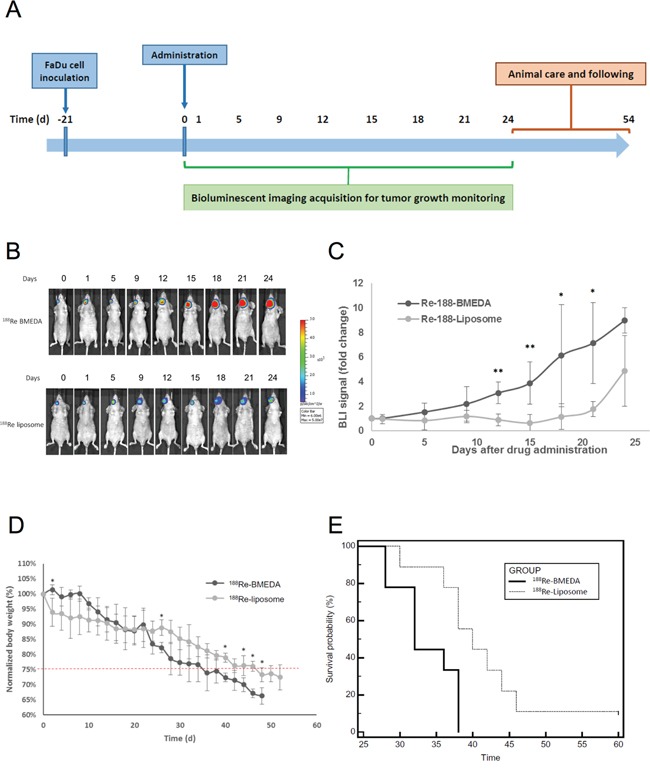
Evaluation of therapeutic efficacy of PEGylated ^188^Re-liposome on HNSCC xenograft tumor model **A.** Illustration of timeline for monitoring the therapeutic efficacy of radiopharmaceuticals on FaDu-3R cells formed tumors using the bioluminescent imaging (BLI); **B.** the ^188^Re-BMEDA (upper panel) and the ^188^Re-liposome (lower panel) were injected with the 80% maximal tolerated dose (MTD, 640 μCi), and the tumor growth was tracked by BLI. (N=9 for each group); **C.** quantification of the tumor bearing mice treated with either ^188^Re-BMEDA or ^188^Re-liposome. The data was normalized to the first BLI acquisition and performed as the fold change. Data are presented as mean ± SD. (*: p<0.05, **p<0.01); **D.** comparison of body weight changes between tumor bearing mice treated with ^188^Re-BMEDA and ^188^Re-liposome; **E.** The survival curves of ^188^Re-BMEDA and ^188^Re-liposome treated mice. The endpoint was set up as the 20% weight loss or death. The results were analyzed by the log-rank test (p < 0.01).

BLI was used to compare the HNSCC growth of tumor-bearing mice that were injected with ^188^Re-liposome or ^188^Re-BMEDA. The timeline of tumor inoculation, drug administration, and tumor imaging time points were illustrated (Figure 4A). The dosages of ^188^Re-liposome and ^188^Re-BMEDA were given at 80% maximum tolerance dose (MTD) with single dose (640 μCi). The BLI signals revealed that the tumor growth was repressed after 12 to 15 days of ^188^Re-liposome administration, and the repression was up to 24 days compared to ^188^Re-BMEDA (Figure 4B). Quantification of the photon signals showed that the tumor growth of the ^188^Re-liposome treated mice was slower than that of ^188^Re-BMEDA ones (Figure 4C). However, it appeared that BLI signals tended to increase in ^188^Re-liposome treated mice after 21 days. This observation was consistent with the expression of Ki-67 marker that was suppressed in xenograft tumors at 5 days but not at 28 days after ^188^Re-liposome treatment (Supplementary Figure 3). Additionally, the body weight of ^188^Re-liposome treated group reduced slower than that of ^188^Re-BMEDA treated group (Figure 4D). The survival of ^188^Re-liposome treated animals was also better than that of ^188^Re-BMEDA treated ones (Figure 4E). These results suggest that ^188^Re-liposome would suppress the growth HNSCC *in vivo*.

### Analysis of the microRNA expressive profiles in the ^188^Re-liposome treated human HNSCC tumors

**Figure 5:**
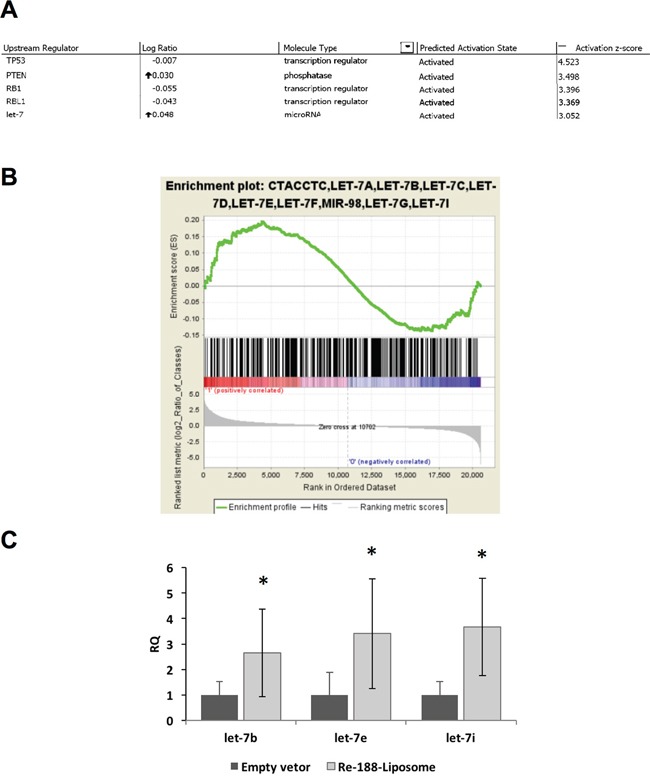
MicroRNA microarray analysis after the HNSCC tumor bearing mouse was treated with PEGylated ^188^Re-liposome **A.** The microarray data were analyzed by IPA, and sorted by the activation z-score >3; **B.** Analysis of microRNA microarray results by GSEA. The *let-7* microRNA family was mostly enriched. (Permutation:1000, sorted by p-value, calculated from the log 2 ratio); **C.** demonstration of *let-7* family members (*let-7b*, *let-7e* and *let-7i*) induced by ^188^Re-liposome but not liposome empty vector using qPCR. Each datum was the mean of four repeats. *: p < 0.05.

Although radiotherapy is known to alter the gene expressive profile of treated tissues, little is known whether ^188^Re-liposome would change the gene expression of tumors after treatment. Here we used the cDNA microarray analysis to investigate the potent signaling pathways that may be stimulated by ^188^Re-liposome. Total RNA was extracted from FaDu-3R tumor treated with ^188^Re-liposome or empty vector (NanoX liposome, Taiwan Liposome Company, Taipei, Taiwan) for 7 days. The gene expressive profile was first subjected to IPA system to identify the potent upstream regulators that could mediate ^188^Re-liposome stimulated gene expression. For z-score over 3, we found that ^188^Re-liposome altered gene expression could be stimulated by several upstream regulators, including *TP53, PTEN* (phosphatase and tensin homolog)*, RB1* (retinoblastoma 1)*, RBL1*(retinoblastoma-like 1) and *let-7* (Figure 5A). These regulators all belongs to tumor suppressor genes. Actually, the complete results of upstream regulators analyzed by IPA also included chemical drugs or inhibitors, but they were not considered in this study (Supplementary Figure 4). Interestingly, *let-7* was the only gene belonging to microRNA family and performing the highest log ratio. To confirm this observation, we exploited the GSEA, a computational approach used to interpret the gene expressive data with common biological characteristics to analyze gene expression controlled by various microRNAs [24]. According to the gene expressive ranking calculated by GSEA, the *let-7* family was identified the most enriched gene set using c3.mir.Gsea gene clusters with the cut-off value of 0.5 log2 ratio by comparing ^188^Re-liposome to empty vectors (Supplementary Figure 5; Figure 5B). The main *let-7* family regulated genes were also listed by the heatmap from GSEA (Supplementary Figure 6). These genes are involved in membrane transport (SLC5A6), cell cycle progression (CDC25A), migration (CCR7), c-Myc activation (MYCBP), and several tumorigenic genes. Furthermore, ^188^Re-liposome induced *let-7* family members, including *let-7b, let-7e* and *let7i*, were confirmed using qPCR (Figure 5C). Thus, the current data provide the evidence that administration of ^188^Re-liposome on HNSCC would increase the *let-7* microRNA expression that leads to suppression of tumorigenesis.

### Knockdown of let-7i compromises ^188^Re-liposome suppressed HNSCC tumor growth *in vivo*

**Figure 6:**
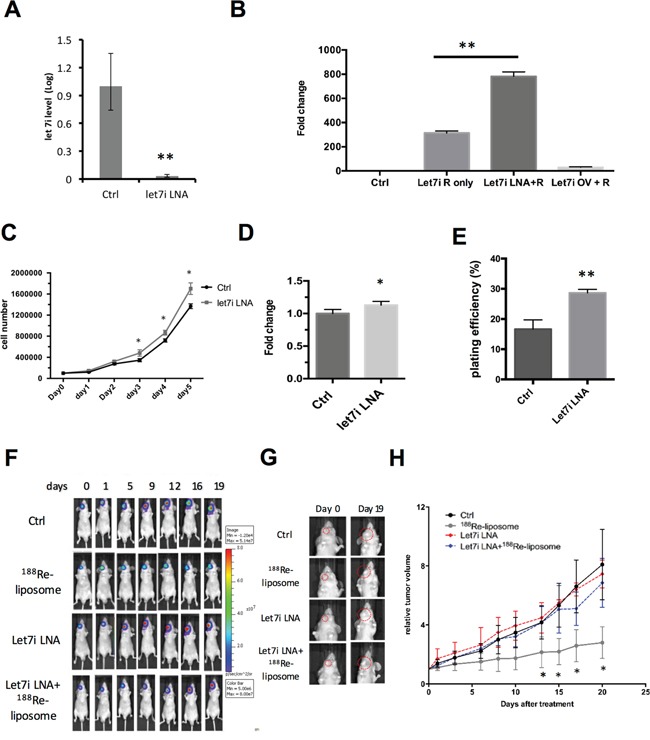
Knockdown of *let-7i* by LNA™ compromises the effects of ^188^Re-liposome on suppressing the growth of human HNSCC tumors *in vivo* **A.** Quantitative PCR analysis for measuring the level of *let-7i* before and after LNA™ transfection; **B.** knockdown of *let-7i* (Let-7i LNA+R) and over-expression of *let-7i* (Let7i OV+R) increased and decreased the luciferase reporter gene activity, respectively. *: p< 0.05; **C.** comparison of cell growth rates with or without transfection of LNA™; **D.** comparison of cell viability in FaDu-3R cells using the MTT assay after transfection of *let-7i* LNA for 48 hours or left untransfected; **E.** comparison of plating efficiency between *let-7i* LNA transfected cells and untransfected cells using the colony formation assay. The colonies were formed and counted after 14 days of incubation (*: p<0.05, **: p<0.01); **F.** BLI imaging for tracking the FaDu-3R tumor growth among control, ^188^Re-liposome treatment, LNA™ treatment and combination of both. Tumors were formed by normal or LNA™ transfected FaDu-3R cells; **G.** visualization of tumor size change under different treatments for 19 days; **H.** tumor growth curves determined by caliperly measuring tumor volume at each time point, and then normalized to the tumor size at day 0. (N=6); *: p < 0.05.

To further investigate whether *let-7* family is essential for the efficacy of ^188^Re-liposome *in vivo*, we used LNA™ to suppress *let-7i* in FaDu-3R cells and then implanted into nude mice for treatment of ^188^Re-liposome in the tumor bearing mice. The qPCR showed that LNA™ could suppress the level of *let-7i* in FaDu-3R cells (Figure 6A). We subsequently used the luciferase reporter gene assay to examine the efficiency of LNA™ on reporter gene expression of pMIR-REPORTER plasmid ablated by *let-7i* (see Materials and Methods). The results showed that LNA™ could enhance, but the *let-7i* over-expressing plasmid could suppress the luciferase activity of pMIR-REPORTER plasmid (Figure 6B). For biological responses, knockdown of *let-7i* increased the cell growth rate of FaDu-3R cells up to 5 days of culturing (Figure 6C). The MTT assay showed that the cell viability of *let7i* LNA transfected cells was slightly higher than that of untransfected cells after 48 hours of transfection (Figure 6D). The colony formation assay further showed that the *let7i* LNA™ transfected cells exhibited stronger colony forming ability than untransfected cells up to 14 days (Figure 6E). Subsequently, we implanted FaDu-3R cells with or without transfection of *let7i* LNA™ into nude mice for tumor formation. Subsequently, the tumor-bearing mice were *i.v.* injected with ^188^Re-liposome or left untreated, followed by BLI examination periodically. The results showed that ^188^Re-liposome could suppress the viability of FaDu-3R tumors, but the effect was reduced by *let7i* LNA™ (Figure 6F). The BLI signals were also reflected on the changes of tumor sizes (Figure 6G). The tumor growth curves apparently showed that ^188^Re-liposome repressed tumor growth could be recovered by *let-7i* LNA™ (Figure 6H). Therefore, these data suggest that *let-7* family is involved in ^188^Re-liposome suppressed HNSCC growth *in vivo*.

## DISCUSSION

Radiopharmaceutical is a critical alternative of radiotherapy because it depends on the drug accumulation in tumor lesion, and specific radionuclide like ^188^Re can emit both particles and γ-rays during decay periods for therapy and diagnosis, so called theranosis [28]. ^188^Re has been conjugated to various materials, including silica coated magnetite and superparamagnetic iron oxide to form nanoparticles for investigating the therapeutic ability in liver cancer cells *in vitro* [29]. For consideration of biological compatibility in clinical, liposome is the most acceptable nanoparticles so far. PEGylated liposome-^188^Re has been applied in various xenograft human tumor models, but not include human HNSCC [8, 9, 30]. The current results revealed that ^188^Re-liposome nanoparticles could also repress xenograft HNSCC tumors and would be a potent theranostic radiopharmaceutical for this tumor type.

FaDu cells are known to induce angiogenesis in nude mice [31]. Thus, it is speculated to be ideal for accumulation of ^188^Re-liposome nanoparticles in the formed tumors via the EPR effect. Indeed, we can easily detect the ^188^Re-liposome signals in orthotopic HNSCC tumors seeded in the buccal cavity using nanoSPECT/CT and Cherenkov luminescent imaging. Interestingly, when FaDu cells were implanted in the tip of tongue reported before [32], we barely detected the accumulation of ^188^Re-liposome in the formed tumor (Supplementary Figure 7). Because FaDu cells belong to hypopharyngeal carcinoma, the microenvironment for tumor formation may influence the subsequent vascular formation and liposomal targeting. These findings suggest that administration of ^188^Re-liposome in different positions of HNSCC in clinical needs to be carefully considered because the therapeutic efficacy may be different.

Despite ^188^Re-liposome can accumulate in the HNSCC tumor lesion, both nanoSPECT/CT imaging and Cerenkov BLI showed non-specific distribution of this radiopharmaceutical *in vivo*. According to the biodistribution analysis, liver and spleen are two major abdomen organs accumulating PEGylated ^188^Re-liposome in this and other report [9]. Although ^188^Re-liposome has not officially entered the clinical trial, a previous report has administrated ^188^Re-hydroxyethylidene diphosphonate (^188^Re–HEDP) with the dose range from 2700 to 3459 MBq (72.9 to 93 mCi) in human prostate cancer treatment [20]. Use of this information, we estimated that the absorbed dose of liver and spleen could be up to 2.493 and 1.809 Gy if ^188^Re-liposome is applied in clinical trial, respectively. Nevertheless, the liver is not a radiosensitive organ because the mean tolerance absorbed dose of liver is 30 Gy [33]. Spleen is also regarded a non-vital organ [21]. The absorbed dose of heart wall in this study was also higher than that of tumor, but the estimated human dose was only 2.057 Gy based on above calculation. This is also far below the mean tolerance absorbed dose (26 Gy) of heart [34]. Taken together, use of ^188^Re-liposome for human HNSCC treatment caused non-specific accumulation in normal tissues should perform lower absorbed dose than their mean tolerance absorbed dose.

Previous study had indicated that 3 Gy absorbed dose of bone marrow may induce up to 1% probability of leukemia within 10 years after IR exposure [35]. In our study, the calculated effective dose of bone marrow was subtotal 0.407 Gy by referring to previous injected dose (3700 MBq) [20]. Therefore, we expected the hematopoietic system may sustain function under ^188^Re-liposome administration. An earlier study revealed that no significant changes in WBC counts were noticed between ^188^Re-liposome and normal saline-treated mice, suggesting no apparent hematotoxicity in ^188^Re-liposome treatment [36]. Furthermore, it has been reported that in normal murine, ^188^Re-liposome did not raise significant death or clinical signs after intravenous injection from 18.5 MBq to 185 MBq [37]. Because only 1.85 MBq was used for tumor-bearing mice in this study, it is speculated that the toxicity of ^188^Re-liposome would be low and acceptable for therapy.

The therapeutic efficacy of ^188^Re-liposome is the primary concern of this study. The results of BLI signals in tumor lesions, body weights and survival curves agree that tumor growth can be suppressed by single injection of ^188^Re-liposome. However, the tumor suppressive effect of ^188^Re-liposome seemed alleviated after three weeks of tracking. This observation suggested that a single dose treatment was not sufficient to eradicate orthotopic HNSCC tumos. Indeed, the IHC staining of Ki67 growth index showed that ^188^Re-liposome can significantly suppress the expression of Ki67 at earlier time but not later (Supplementary Figure 3), suggesting that ^188^Re-liposome may cause cytostatic effect rather than cytotoxic effect. Because FaDu cells are regarded radioresistant HNSCC [38], multi-dosage or adjuvant chemo-radiotherapy may enhance the therapeutic efficacy of ^188^Re-liposome on this cell type.

In this study, the gene expressive profile of HNSCC orthotopic tumor after ^188^Re-liposome treatment was further investigated to better understand the biological mechanisms of this radiopharmaceutical. The cDNA microarray data were analyzed for potent activated upstream regulators by calculating the activation z-score representing the bias in gene regulation according to the instruction of IPA. Although the z-score filtered at 2.0 was regarded activated, we set at 3.0 to focus on more responsive upstream regulators. Under this condition, TP53 and PTEN were found to correlate to the expression of downstream genes after ^188^Re-liposome treatment. This result agrees previous reports that p53 and PTEN are important for apoptosis or tumor suppression by radiotherapy [39, 40]. Involvement of RB1 and RBL1 as potent major upstream regulators suggests that ^188^Re-liposome would influence the cell cycle progression. Surprisingly, *let-7* showed high z-score with apparent up-regulation, and this is the only microRNA family found by IPA, even the z-score was filtered at 2.0 (data not shown). Further analysis using GSEA showed that among various microRNAs, *let-7* microRNA family was the mostly enriched gene set in ^188^Re-liposome treated group compared to the control. Although we showed that *let-7* could be induced by ^188^Re-liposome, little is known whether the expression of p53, PTEN and RB will be directly activated by this radioactive compound to suppress tumor growth. It is important to compare the effects of these tumor suppressor genes on the therapeutic efficacy of ^188^Re-liposome in the future.

Knockdown of *let-7i* also decreased the effects of ^188^Re-liposome on suppressing the growth of HNSCC xenograft tumors. Because here we only used the *let-7i* LNA™, it is not excluded that concomitantly knockdown of other *let-7* members would further suppress the therapeutic efficacy of ^188^Re-liposome. Recently, *let-7i* has been reported to be repressed coordinately by Twist-1 and BMI-1 [26]. Here we also attempted to investigate the expressive levels of Twist1 and BMI1 in ^188^Re-liposome treated HNSCC tumor using real-time qPCR. Although BMI-1 showed no significant decrease, TWIST-1 expression was greatly down-regulated by ^188^Re-liposome compared to NanoX liposome empty vector (Supplementary Figure 8). Although Twist-1 can cooperate with BMI-1 to repress *let-7i*, BMI-1 can also independently repress *let-7i* through the polycomb complex-dependent mechanism [41]. Therefore, ^188^Re-liposome may specific suppress TWIST-1 signaling pathway but not BMI-1 pathways to regulate *let-7* biogenesis. How ^188^Re-liposome can distinguish these signaling pathways to induce *let-7* expression is of interest to further investigate.

In summary, the current data demonstrated that ^188^Re-liposome could efficiently repress human HNSCC in the orthotopic xenograft tumor model. The measured biodistribution, pharmacokinetics, and calculated organ dosimetry suggest that the safety of ^188^Re-liposome should be acceptable. ^188^Re-liposome could change the gene expressive profile of tumor. This phenomenon was associated with several well-known tumor suppressor genes that acted as potent upstream regulators activated by ^188^Re-liposome. Interestingly, the *let-7* family was the only microRNA involved in the therapeutic efficacy of ^188^Re-liposome. To the best of our knowledge, this is the first report showing that ^188^Re-liposome can induce *let-7* expression in HNSCC tumors. Since the use of ^188^Re-liposome has entered the clinical trial phase I in Taiwan (1002001INER085), comprehension of the molecular mechanisms induced by this radiopharmaceutical would be benefit for development of precise personal therapy in the future. Taken together, ^188^Re-liposome would be a novel approach for radio-chemotherapy of human HNSCC via induction of tumor suppressive signaling pathways.

## MATERIALS AND METHODS

### Cell lines

Human hypopharyngeal carcinoma FaDu cells (American Type Culture Collection, Manassas, VA, USA) and FaDu-3R cells harboring multiple reporter genes were maintained in RPMI-1640 (Life Technologies Inc., Carlsbad, CA, USA) with supplement of 10% fetal bovine serum (Thermo Fisher Scientific Inc., Waltham, MA, USA). The 293T cells (CRL-3216, American Type Culture Collection, Manassas, VA, USA) were cultured in Dulbecco's modified eagle medium (DMEM, Life Technologies Inc., Carlsbad, CA, USA) with 10% fetal bovine serum supplied. Cells were incubated at 37°C in a humidified incubator (Thermo Fisher Scientific Inc., Waltham, MA, USA) containing 5% CO_2_.

### Preparation and quality control of ^188^Re-liposome

The manufacturing procedures of ^188^Re-liposome and decoration of liposome using PEG have been described previously [9]. The quality validation including particle size (84.6 ± 4.12nm) and surface charge (1.1 ± 1.9mV) were measured by the dynamic light scattering. The N, N-bis(2-mercaptoethyl)-N9, N9-diethylethylenediamine (BMEDA) chelator conjugating to Na^188^ReO_4_ (^188^Re-BMEDA) was used to compare the analytic results of ^188^Re-liposome *in vivo*.

### Transduction of multicistronic reporter genes

The LT-3R multicistronic lentiviral plasmid contains three reporter genes, including green fluorescent protein (GFP) gene, firefly luciferase gene (fLuc) and herpes simplex virus type 1-thymidine kinase (HSV1-tk) gene [19]. This plasmid was used to establish FaDu-3R stable cells for screening and reporter gene imaging. Transduced cells were screened using the fluorescence-activated cell sorting (FACS, FACSAria, BD Biosciences, San Jose, CA, USA) to isolate the stable cells.

### Western blot analysis

The protein extraction, gel running and Western blot analysis for detecting the expression of HSV1-tk were followed based on our previous report [20].

### Human HNSCC tumor-bearing animal model

The stable cell line with multiple reporters, namely FaDu-3R cells, were inoculated on Balb/C nude mice with orthotopic injection. The FaDu-3R cells were carefully cultured and resuspended in serum-free RPMI-1640 (Life Technologies Inc., Carlsbad, CA, USA) at a concentration of 10^6^ cells/50 μl. The mice were anesthetized by ketamine/xylazine mixture and placed vertically. The mouth was opened and stabilized with handhold tweezer. The 29G needle syringe (Terumo Co., Tokyo, Japan) was then inserted for 5 mm and injected through the outer muscle of lower jaw on the right side of mice. Mice were quickly released and cared with heat lamp until awaken. For tongue tumor-bearing model, the mice were anesthetized and placed supine. Mouth was opened by surgical tools and the tongue was pulled out of the oral cavity. Cells were resuspended and injected by the 29G insulin syringe directly on the tip of tongue. Mice were promptly placed in prone position and cared until awaken. To detect the accumulation of ^123^I-5-iodo-2′-fluoro-1-β-D-arabinofuranosyluracil (^123^I-FIAU) or ^188^Re-liposome in FaDu-3R tumor-bearing mice, the nanoSPECT/CT scanner was used (Mediso Ltd. Alsotorokvesz, Budapest, Hungary). All the experimental animals were housed and cared by following the standard protocols laid by the National Laboratory Animal Center, and approved by the Institutional Animal Care and Use Committee (IACUC) of National Yang-Ming University.

### Bio-distribution and pharmacokinetic analysis

Forty HNSCC tumor-bearing mice (5 mice per group) were intravenously injected with 1.85MBq/100 μl ^188^Re-BMEDA or ^188^Re-liposome separately. For biodistribution analysis, mice were sacrificed by CO_2_ asphyxiation to collect organs and tumors after 1, 4, 24, and 48 hours of injection. Collected organs were weighted and counted by the γ-counter (Cobra II Auto-Gamma Counter, PerkinElmer Inc., Waltham, MA, USA). The results were presented as percentage injected dose per gram (%ID/g). The statistic results were performed with the means ± standard deviations. For pharmacokinetic analysis, the blood samples were collected by the tail vein puncture with microliter capillary tubes at different time points, including 0.083, 0.25, 0.5, 1, 2, 4, 8, 12, 16, 20, 24, 28, 32, 36, 40, 44, 48, 52, and 56 hours. The samples were counted and calculated by Pharsight WinNonlin 5.2 software (Certara L.P., St. Louis, MO, USA), and the noncompartment (NCA) model 201 was selected for analysis.

### Dosimetric evaluation of ^188^Re-liposome absorbed radiation dose *in vivo*

The dosimetry of percentage injected dose activity per weight unit (% ID/g) in human was extrapolated from murine bio-distribution data following the previous report [21]. The organ absorbed doses were then calculated using the guideline of Medical Internal Radiation Dosimetry (MIRD) pamphlets implanted in the OLINDA/EXM software [22, 23]. For tumor absorbed dose, the number of disintegration was calculated and input to the sphere modeling of OLINDA/EXM software to estimate the absorbed dose of tumor in each spheroid mass.

### Evaluation of therapeutic efficacy of ^188^Re-liposome on HNSCC xenograft tumors

The therapeutic efficacy was assessed by the bioluminescent signal acquired from the *In Vivo* Imaging System (IVIS 50, Perkin Elmer Inc., Waltham, MA, USA) or caliper measurement of tumor volume. Tumor volume was calculated by the formula as (width^2^ x length)/2. the quantified results were normalized to the day treatment begun. The survival was recorded since the first mice died from the treatment or the disease up to 60 days. The record was then calculated by MedCalc software with the log-rank examination for the significant difference. The Kaplan-Meier plot was used for demonstration of survival with 95% confidence interval.

### Gene expression microarray analysis

The xenograft tumors were dissected from the orthotopic sites and washed by buffer saline twice. The tissues were diced into small pieces and flash frozen by liquid nitrogen and subjected to be grounded into fine powders. The total RNA of the tissues were extracted using TRIzol reagent (Life Technologies Inc., Carlsbad, CA, USA) following the manufacturer's instructions. The extracted total RNA was subjected to the Human Genome Array U133 plus 2 Chip (Affymetrix, Santa Clara, CA, USA) for microarray analysis (National Microarray & Gene Expression Analysis Core Facility of the National Research Program for Genomic Medicine, Taipei, Taiwan). The microarray data were uploaded to the web-based Ingenuity Pathway Analysis system (IPA, Qiagen, Hilden, Germany) for analysis of gene expression. For gene set enrichment analysis (GSEA, Broad Institute, Cambridge, MA, USA) [24]. GSEA 2.1.0 software was used for the calculation with the c3.mir.v4.0.motif.gmt geneset with 1000 numbers of permutation. The results were optimized by eliminating the false discovery rate by 25%.

### MicroRNA targets prediction and in silico investigation

The target prediction of microRNA was achieved by overall online search with DIANA-microT (http://diana.imis.athena-innovation.gr/DianaTools/index.php), TargetScan (http://www.targetscan.org/), PicTar (http://pictar.mdc-berlin.de/cgi-bin/new_PicTar_vertebrate.cgi), and microRNA.org (http://www.microrna.org/microrna/home.do) databases. All the targets were concluded and the overlapped targets was then screened by score ranking.

### Real-time quantitative polymerase chain reaction (qPCR)

RNA was extracted using the TriZol reagent and then extracted using the Zymo spin column (Zymo Research Co., Irvine, CA, USA). The total RNA was quantified using the NanoDrop Spectrophotometer (Thermo Fisher Scientific Inc., Waltham, MA, USA). Adequate amount of total RNA was reversely transcribed to complementary DNA (cDNA) using the SuperScript III reverse transcriptase (Life Technologies Inc., Carlsbad, CA, USA). For mRNA, the oligo dT primer was used for the reverse transcription. For various microRNAs, the stem loop gene specific primers were used separately (Supplementary Table 1). The qPCR was performed by the StepOne Plus Real-time PCR system (Life Technologies Inc., Carlsbad, CA, USA) with the Kapa Fast SYBR Green Master Mix (Kapa Biosystems, Wilmington, MA, USA). The quantification analysis was followed according to the instructions of the StepOne Plus software 2.1 (Applied Biosystems Inc., Carlsbad, CA, USA).

### Luciferase reporter gene assay for let-7i activity

The luciferase assay was conducted according to a previous report with slight modifications [25]. Twenty nM chemically modified hsa-*let-7i*-5p miRCURY locked nucleic acid (LNA™, Exiqon, Los Angeles, CA, USA) specifically targeting on *let-7i* (5′-ACAGCACAAACTACTACCTC-3′) was transfected into cells using the JetPEI transfection reagent (Polyplus-transfection, SA, Illkirch, France). To determine the effect of LNA™, the pMIR-REPORTER plasmid for *let-7i* targeting (a gift kindly provided by Dr. Muh-Hwa Yang in National Yang-Ming University) was used for co-transfection with LNA™ [26].

### Cell viability assay

The cell viability was determined using 3-(4,5-cimethylthiazol-2-yl)-2,5-diphenyl tetrazolium bromide (MTT) assay and colony formation assay. In brief, FaDu cells were seeded in a 96-well plate (4000 cells per well) after transfected with LNA™ for 48hrs, and incubated at 37°C for 2 days. After removal of the supernatant, 1mg/ml MTT solution (Sigma-Aldrich Co., St. Louis, MO, USA) was mixed with serum-free medium and added to each well followed by dimethyl sulfoxide to dissolve crystals. The plate was then detected at 570 nm wavelength using the ELISA reader (ELISA plate reader; Bio-Tek Instruments, Winooski, VT, USA). For colony formation assay, 100 cells were seeded in 6-cm dishes after transfected with LNA™ for 48hrs, and incubated at 37°C for 2 weeks without disturbance. Formed colonies were first fixed by 4% formaldehyde and then visualized by staining with crystal violet solution.

### Statistical analysis

The results demonstrated in this study was analyzed by Student's t-test, and p < 0.05 was determined as significant difference. The Kaplan-Meier curves was plotted by the MedCalc (MedCalc Software, Ostend, Belgium) integrated program, and the results were analyzed by the log-rank test.

## SUPPLEMENTARY MATERIALS FIGURES AND TABLE


