# Supplementary material for: Involvement of let-7 microRNA for the therapeutic effects of Rhenium-188-embedded liposomal nanoparticles on orthotopic human head and neck cancer model
Source: Oncotarget. 2016 Aug 29;7(40):65782–96. doi: 10.18632/oncotarget.11666 (PMC5323192; doi:10.18632/oncotarget.11666)
Supplement: Supplementary file 1 [file oncotarget-07-65782-s001.pdf]

Involvement of *let-7* microRNA for the therapeutic effects of Rhenium-188–embedded liposomal nanoparticles on orthotopic human head and neck cancer model

SUPPLEMENTARY FIGURES AND TABLE

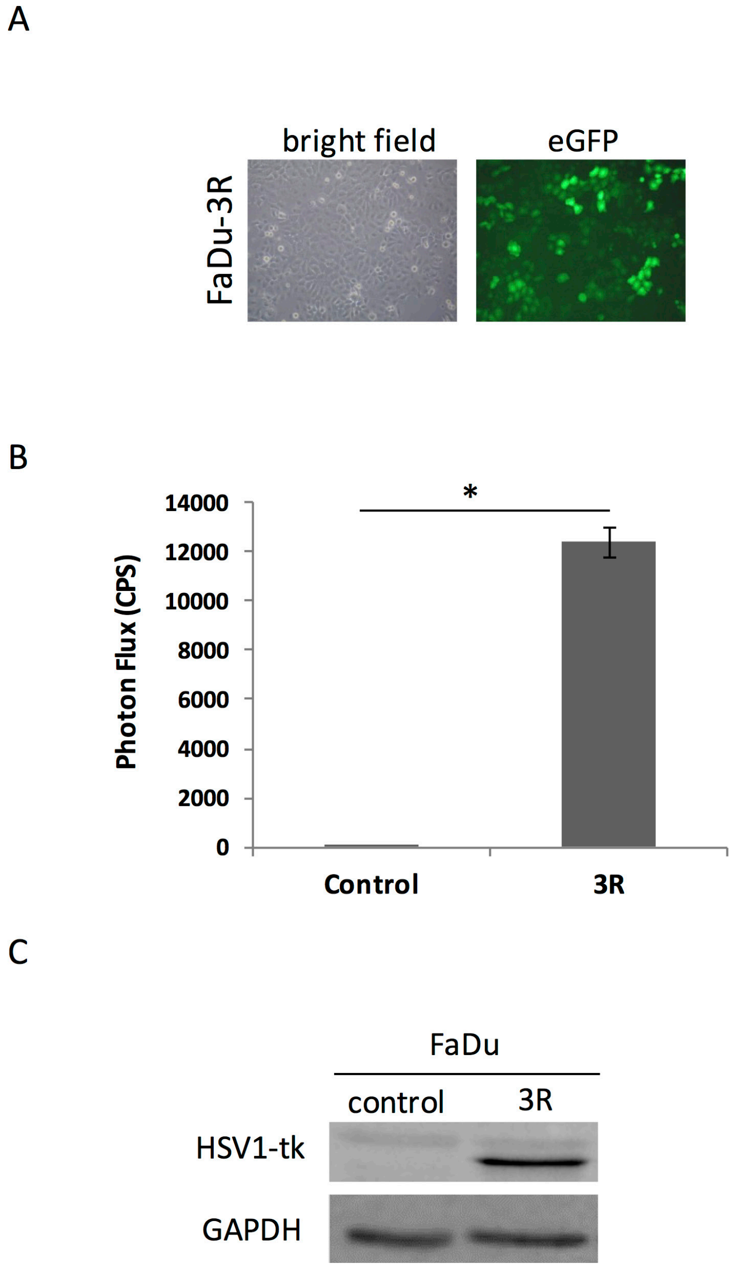

Supplementary Figure S1: Establishment of FaDu-3R cells. A. Detection of GFP expression using the fluorescent microscope; **B.** detection of luciferase activity using the luciferase assay; **C.** detection of HSV1-tk expression using anti-HSV1-tk antibody.

Doses from Nuclide : Re-188 in Sphere	
Sphere Mass (g)	Dose Mass (mGy/MBq)
0.01	1.39 E001
0.1	2.71 E000
0.5	6.70 E-01
1.0	3.56 E-01
2.0	1.86 E-01
4.0	9.64 E-02
6.0	6.53 E-02
8.0	4.96 E-02
10.0	3.98 E-02
20.0	2.03 E-02
40.0	1.03 E-02
60.0	6.95 E-03
80.0	5.24 E-03
100.0	4.19 E-03
300.0	1.42 E-03
400.0	1.07 E-03
500.0	8.56 E-04
600.0	7.16 E-04
1,000.0	4.33 E-04

Supplementary Figure S2: Correlation between the size of spheroid tumor mass and the estimated absorbed dose.

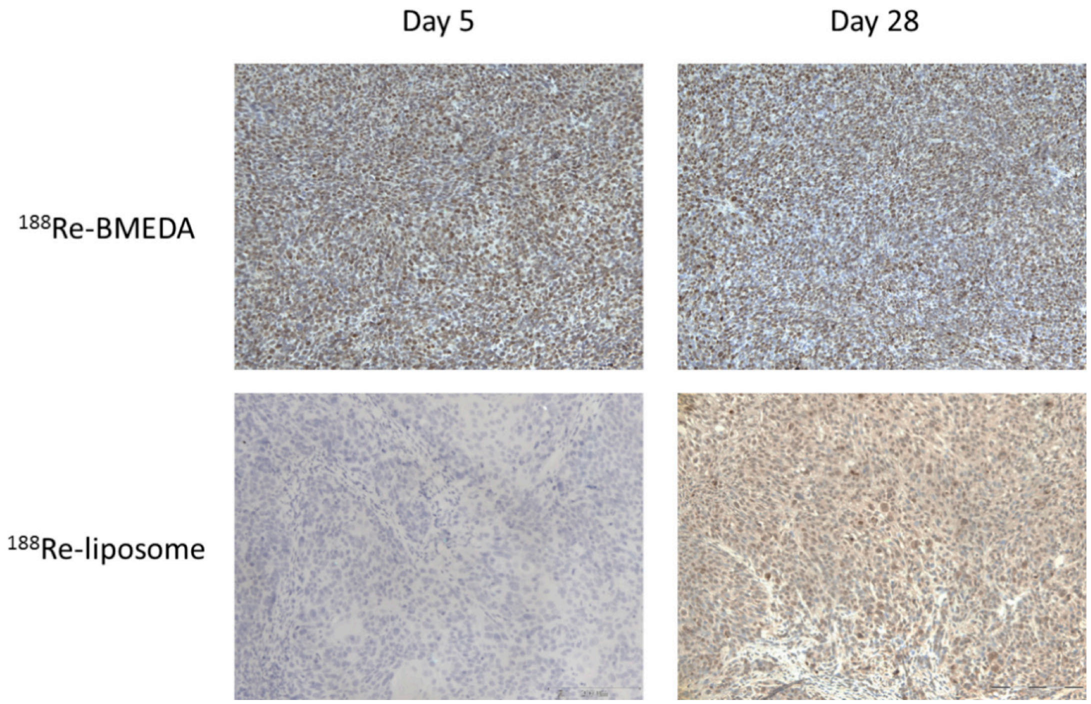

Supplementary Figure S3: The immunohistochemistry sections were stained with Ki67 to perform as the growth index of tumor. The fields were randomly chosen and captured by the microscopy by 10X objective.

Upstream Regulator	Exp Log Ratio	Molecule Type	Predicted Activatio...	Activation z-score	p-value of overlap	Target molecu...	Mechanistic N...
TP53	↓-0.021	transcription regulator	Activated	4.523	7.53E-12	↑ABHD4, ↓AEN ...all 108	205 (19)
PTEN	↑0.019	phosphatase	Activated	3.498	4.49E-04	↓ABCC4, ↑ABTB1 ...all 32	245 (17)
U0126		chemical - kinase inhibi...	Activated	3.470	4.25E-09	↓ANGPTL4, ↓... ...all 48	319 (22)
fulvestrant		chemical drug	Activated	3.458	2.45E-09	↓BLM, ↑CCDC80 ...all 32	209 (18)
RB1	↓-0.018	transcription regulator	Activated	3.396	8.51E-08	↓BLM, ↓CCNA2, ...all 31	190 (13)
isotretinoin		biologic drug	Activated	3.370	8.29E-10	↑ABCA1, ↑AN... ...all 18	306 (22)
RBL1	↓-0.039	transcription regulator	Activated	3.369	4.16E-06	↓CCNA2, ↓CC... ...all 13	251 (20)
PD98059		chemical - kinase inhibi...	Activated	3.195	7.21E-12	↑ABCA1, ↑ATP... ...all 59	284 (22)
lenalidomide		chemical drug	Activated	3.153	2.55E-05	↑BCL11B, ↓DL... ...all 17	
let-7	↓-0.034	microrna	Activated	3.052	9.18E-11	↓CCNA2, ↓CCNJ ...all 25	202 (13)
BMS-690514		chemical drug	Activated	3.000	5.23E-06	↑CTSF, ↑DDIT3, ·. ...all 9	
Rb		group	Activated	2.970	1.29E-04	↓CCNA2, ↓CC... ...all 11	184 (15)
SMARCA4	↓-0.016	transcription regulator	Activated	2.795	1.70E-06	↑ABCA1, ↑ATP... ...all 32	226 (20)
dexamethasone		chemical drug	Activated	2.772	5.58E-12	↑ADHFE1, ↑... ...all 122	300 (18)
estrogen receptor		group	Activated	2.770	6.90E-08	↑ANXA9, ↓CALB2 ...all 29	264 (20)
CDKN2A	↑0.060	transcription regulator	Activated	2.728	1.16E-05	↓BLM, ↓CCNA2, ...all 24	207 (16)
SFTPA1	↓-0.014	transporter	Activated	2.714	2.14E-04	↑AMOT, ↓ANG... ...all 11	203 (12)
calcitriol		chemical drug	Activated	2.657	8.46E-16	↑CASR, ↓CCNA2 ...all 59	308 (23)
WISP2	↓-0.038	growth factor	Activated	2.630	4.84E-04	↑CD24, ↓CD44, ·. ...all 7	106 (7)
CDH1	↑0.028	other	Activated	2.619	1.87E-02	↓CD44, ↓FOSL1, ...all 7	
MITF	↓-0.032	transcription regulator	Activated	2.588	1.09E-02	↑APOLD1, ↑C... ...all 13	
tretinoin		chemical - endogenous ...	Activated	2.548	7.32E-08	↑ABCA1, ↓ABCE1 ...all 98	298 (23)
pimagedine		chemical drug	Activated	2.433	3.26E-03	↓COL4A1, ↓CXCL8 ...all 6	119 (7)
brefeldin A		chemical - endogenous ...	Activated	2.415	8.96E-03	↑ABCA12, ↓CD44 ...all 6	203 (12)
Calcineurin protein(s)		complex	Activated	2.381	5.85E-03	↑CBLB, ↑CYGB, ↓.. ...all 7	250 (24)
RBL2	↑0.032	other	Activated	2.369	6.68E-03	↓ABCF2, ↓CCNE1 ...all 10	219 (19)
epigallocatechin-gallate		chemical drug	Activated	2.352	7.44E-02	↓CCL22, ↓CCL5 ...all 14	
bexarotene		chemical drug	Activated	2.345	1.76E-02	↑ABCA1, ↑AKR1C3 ...all 9	
HDAC2	↓-0.053	transcription regulator	Activated	2.333	4.12E-05	↑BCL6, ↑CCNG2 ...all 15	249 (20)
FOXO3	↓-0.027	transcription regulator	Activated	2.294	8.19E-07	↑ALDH3A1, ↑... ...all 22	261 (20)
HDAC1	↓-0.008	transcription regulator	Activated	2.252	6.81E-07	↑BCL6, ↓CCL5, ·. ...all 25	178 (17)
TNFSF13	↑0.024	cytokine	Activated	2.236	2.82E-03	↑BCL6, ↓CDC6, ·.. ...all 6	
BNIP3L	↑0.092	other	Activated	2.236	7.56E-02	↓CCNA2, ↓GSG2, ...all 5	
SOCS3	↑0.003	phosphatase	Activated	2.236	1.90E-01	↑ABCA1, ↓EGR1, ...all 5	
PP2/AG1879 tyrosine kinase i		chemical - kinase inhibi...	Activated	2.216	1.62E-01	↓CXCL8, ↓FOS, ↓.. ...all 5	
DACH1	↑0.022	transcription regulator	Activated	2.213	3.26E-03	↓CDC25A, ↓EGR1 ...all 6	180 (10)
PD 153035		chemical drug	Activated	2.200	1.38E-03	↓CXCL8, ↑DDIT3 ...all 5	259 (18)
HNF1A	↑0.011	transcription regulator	Activated	2.173	1.12E-03	↑ACE2, ↑ADH1A ...all 32	119 (2)
medroxyprogesterone acetate		chemical drug	Activated	2.158	1.27E-01	↓CCL5, ↓CD44, ·.. ...all 8	
IFN Beta		group	Activated	2.150	5.24E-01	↓CXCL8, ↑IL12A, ...all 5	
Interferon alpha		group	Activated	2.119	6.21E-02	↑A4GALT, ↓C... ...all 20	
BMP2	↑0.019	growth factor	Activated	2.076	1.79E-01	↑BPIFB3, ↑EPHX2 ...all 11	
wortmannin		chemical - kinase inhibi...	Activated	2.051	1.13E-02	↓CCL5, ↓CD44, ...all 15	
Calmodulin		group	Activated	2.000	8.53E-02	↓CD44, ↑EPHB6, ...all 5	
TOB1	↑0.018	transcription regulator	Activated	2.000	1.21E-01	↓CCNA2, ↓CDT1 ...all 4	
NLRP3	↑0.015	other	Activated	2.000	1.91E-01	↓ESPL1, ↓PRKDC ...all 4	
bisindolylmaleimide		chemical - kinase inhibi...	Activated	2.000	2.08E-02	↓CXCL8, ↓EGR1, ...all 4	

Supplementary Figure S4: Results of IPA analysis for upstream regulators that were involved in regulating ^{188}Re-liposome caused gene re-expression in HNSCC tumors.

NAME	SIZE	ES	LEADING EDGE
CTACCTC,LET-7A,LET-7B,LET-7C,LET-7D,LET-7E,LET-7F,MIR-98,LET-7G,LET-7I	337	0.19612668	tags=17%, list=21%, signal=21%
TGGTGCT,MIR-29A,MIR-29B,MIR-29C	441	0.15790977	tags=15%, list=21%, signal=18%
CACTGCC,MIR-34A,MIR-34C,MIR-449	256	0.20749177	tags=20%, list=23%, signal=26%
TACGGGT,MIR-99A,MIR-100,MIR-99B	21	0.14401923	tags=43%, list=40%, signal=71%
ACATTCC,MIR-1,MIR-206	252	0.19843787	tags=17%, list=23%, signal=21%
AAGTCCA,MIR-422B,MIR-422A	55	0.24517448	tags=31%, list=28%, signal=43%
AAAGGGA,MIR-204,MIR-211	197	0.13499105	tags=14%, list=20%, signal=18%
CACTGTG,MIR-128A,MIR-128B	284	0.26406452	tags=15%, list=18%, signal=19%
ACTGAAA,MIR-30A-3P,MIR-30E-3P	163	0.1394582	tags=11%, list=20%, signal=14%
CTCAGGG,MIR-125B,MIR-125A	281	0.17558943	tags=21%, list=26%, signal=28%
GTATGAT,MIR-154,MIR-487	55	0.15767176	tags=7%, list=13%, signal=8%
CAGTGTT,MIR-141,MIR-200A	268	0.20007987	tags=19%, list=24%, signal=25%
AGTTCTC,MIR-146A,MIR-146B	50	0.22212783	tags=28%, list=27%, signal=38%
ATGTAGC,MIR-221,MIR-222	112	0.29823235	tags=6%, list=5%, signal=7%
ACTGTGA,MIR-27A,MIR-27B	387	0.20290704	tags=14%, list=20%, signal=18%
CACTTTG,MIR-520G,MIR-520H	204	0.17559552	tags=11%, list=17%, signal=13%
GGGACCA,MIR-133A,MIR-133B	164	0.24341387	tags=20%, list=20%, signal=25%
AAGCCAT,MIR-135A,MIR-135B	282	0.19822319	tags=15%, list=21%, signal=18%
ATGCTGC,MIR-103,MIR-107	186	0.27584177	tags=17%, list=18%, signal=20%
ACAGGGT,MIR-10A,MIR-10B	106	0.19831675	tags=14%, list=17%, signal=17%
GGCCAGT,MIR-193A,MIR-193B	83	0.25609466	tags=18%, list=22%, signal=23%
TCTGATC,MIR-383	39	0.36142987	tags=10%, list=9%, signal=11%
AGTCAGC,MIR-345	51	0.19630477	tags=16%, list=22%, signal=20%
GTGTGAG,MIR-342	59	0.19974932	tags=17%, list=22%, signal=22%

Supplementary Figure S5: Gene set enrichment analysis (GSEA) concluded the ranking of highly regulated microRNA gene sets.

FaDu_Re	FaDu_NC		FaDu_Re	FaDu_NC													
								PDGFB			CLDNI12			GAN			ADAMTS5
								DCLREIB			MAPK6			NME4			LRRC1
		Sample Name			Sample Name			SLC25A18			GABBR2			TEAD3			HAND1
		PYY2			SLC1GA14			CHRD			CPA4			SEMA4C			KCTD17
		CECR6			TMEM110			NKD1			DVL3			PGM2L1			RPUSD3
		PLA2G3			SLC6A1			SOCS4			TMPRSS2			PAK1			CBEA2T3
		ARHGAP20			GNG5			SGCD			PCYT1B			EGR3			LRIG3
		DDX19B			ADRB2			HMGA2			CRY2			EZH2			PS8
		LIMD2			MYCBP			FRAS1			APBB3			ETNK2			OSMR
		ZNE740			PCGF3			ARHGAP28			RICTOR			ADAM15			TAF9B
		ANKRD43			PIGA			STK40			RUFY3			NLK			PBX2
		TNERSF1B			AECCIO			ZNE583			SYT7			FZD4			LRIG1
		COL24A1			KLHDC8B			PARD6B			WDR37			TBKBP1			USP6
		ARID3B			ULK2			DCUN1D3			GNAL			FIGN			NIPA1
		PLEKHG6			M1B1			CYP19A1			RASL1OB			PRRX1			APIS1
		SLAMF6			KLE9			CPSF4			SNX16			RAB15			MXD1
		DDN			RGS16			OSBPL3			CDH22			SYT11			SEMA4G
		ADRB3			STX17			HMGA1			LSM11			PHF8			OPRM1
		PCDHI9			ZBTB39			PLCXD3			DKK3			SLC25A24			MASP1
		SNAI3			E2F6			DLAPH2			NDST2			ARHGEF15			DLGAP4
		SLC8A2			FN1P1			BCL2L1			MGAT4A			ITGB3			RAB4OC
		DCX			ERGIC1			CBX2			ZNE354A			HSPAI4			CPM
		DCUN1D2			MYO1F			CACNG4			NSMCE2			EDA			SLC26A9
		PRTG			SMARCAD1			KIAA0664			CDC25A			RALB			HOXD1
		NEASC			ACVR1B			SLC5A6			ARRDC4			DDXI9A			E2F5
		ILI3			PLD3			CCR7			SCN4B			CPEB2			GCNT4
		ACSL6			AAK1			ATP2B3			SLC25A27			SLC2OA1			CYP46A1
		TBX5			GGA3			COL1A1			NXT2			MEF2D			PPP1R12B
		CLASP2			MECP2			RPUSD2			CCNJ			SCUBE3			B3GNT6
		HIC2			TMEM2			BIN3			ZFYVE26			ATP2B1			PTPRU
		COLI4A1			IRS2			HOXC11			ZNE318			RASGPP1			DOCK3
		SEMA4F			UNC5A			DTX2			KLHLI3			ANKRD28			SCN5A
		ANKFY1			WNT1			LOORL			TRIM67			MDE1			RSPO2
		NOVA1			HOXA1			MFD8			DHX57			PLXND1			

Supplementary Figure S6: List of genes affected by *let-7* family based on GSEA.

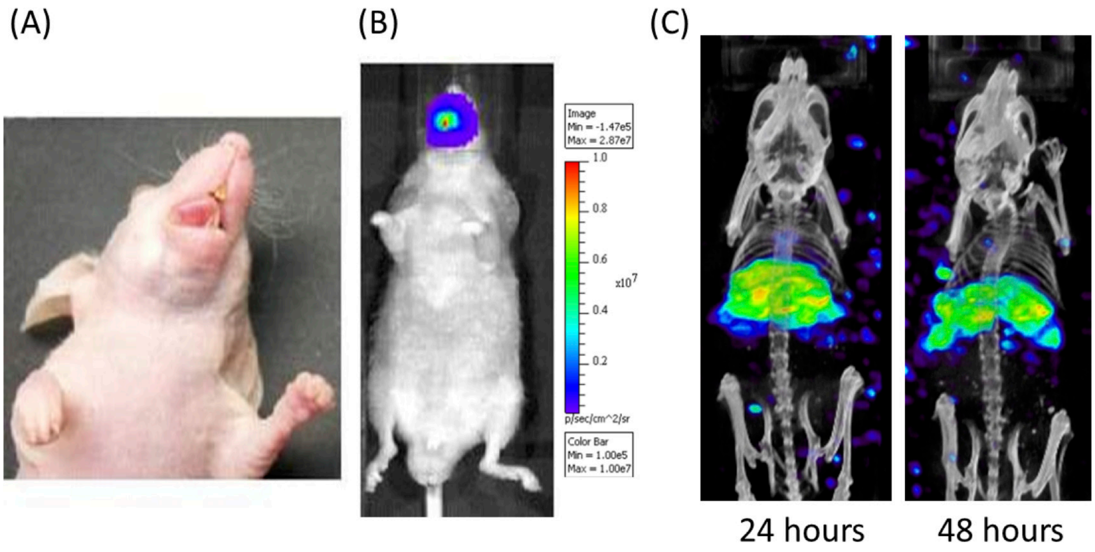

Supplementary Figure S7: The establishment of orthotopic HNSCC by different approach. A. The cells were injected on the tip of tongue; **B.** the tumor formation was monitored by BLI; **C.** i.v. injection of ^{188}Re-liposome showed no accumulation in tumor lesion up to 48 hours.

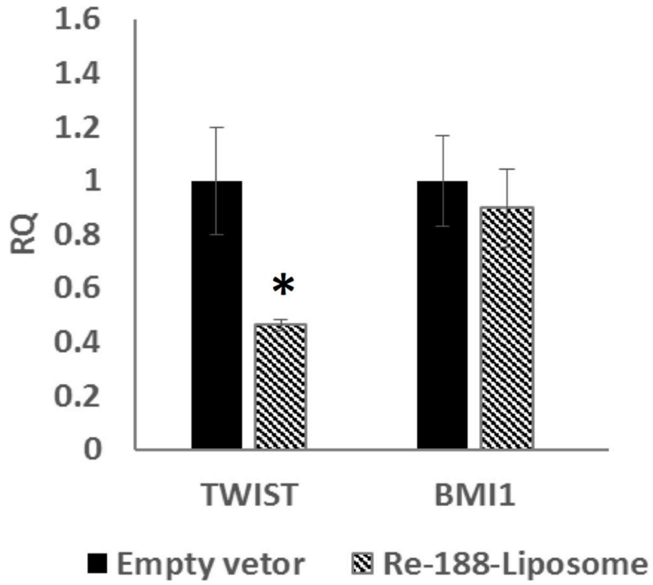

Supplementary Figure S8: Down-regulation of Twist-1 but not BMI-1 by ^{188}Re-liposome to affect *let-7* expression using qPCR.

Supplementary Table S1: The primer sets used for qPCR

Primer id	sequence 5' to 3'
BMI1_F	ggAgACCAgCAAgTATTgTCCTTTTg
BMI1_R	CATTgCTgCTgggCATCgTAAg
TWIST_F	AgCTACgCCTTCTCggTCT
TWIST_R	CCTTCTCTggAAACAATgACATC
β-actin_F	ggAAATCgTgCgTgACATTAAg
β-actin_R	ggCCATCTCTTgCTCgAAgT
U6_F	CgCTTCggCAgCAgCACATATAC
U6_R	TTCACgAATTTgCgTgTCAT
let-7b stemloop	gTCgTATCCAgTgCAgggTCCgAggTATTCgCACTggATACgACAACCAC
*let-7b*_F	gCCgCTTgAggTAgTAggTTgT
let-7e stemloop	gTCgTATCCAgTgCAgggTCCgAggTATTCgCACTggATACgACAACTAT
*let-7e*_F	gCCgCTTgAggTAggAggTTgT
let-7i stemloop	gTCgTATCCAgTgCAgggTCCgAggTATTCgCACTggATACgACAACAgC
*let-7i*_F	gCCgCTTgAggTAgTAgTTTgT
Universal_R	CCAgTgCAgggTCCgAggT
